# The Alcohol Flushing Response: An Unrecognized Risk Factor for Esophageal Cancer from Alcohol Consumption

**DOI:** 10.1371/journal.pmed.1000050

**Published:** 2009-03-24

**Authors:** Philip J Brooks, Mary-Anne Enoch, David Goldman, Ting-Kai Li, Akira Yokoyama

## Abstract

Philip Brooks and colleagues discuss evidence linking the alcohol flushing response (predominantly due to ALDH2 deficiency) with a much higher risk of esophageal cancer from alcohol consumption.

Approximately 36% of East Asians (Japanese, Chinese, and Koreans) show a characteristic physiological response to drinking alcohol that includes facial flushing (see Figure 1), nausea, and tachycardia [1] . This so-called alcohol flushing response (also known as “Asian flush” or “Asian glow”) is predominantly due to an inherited deficiency in the enzyme aldehyde dehydrogenase 2 (ALDH2) [2]. Although clinicians and the East Asian public generally know about the alcohol flushing response (e.g., http://www.echeng.com/asianblush/), few are aware of the accumulating evidence that ALDH2-deficient individuals are at much higher risk of esophageal cancer (specifically squamous cell carcinoma) from alcohol consumption than individuals with fully active ALDH2. This is particularly unfortunate as esophageal cancer is one of the deadliest cancers worldwide [3], with five-year survival rates of 15.6% in the United States, 12.3% in Europe, and 31.6% in Japan [4].

**Figure 1:**
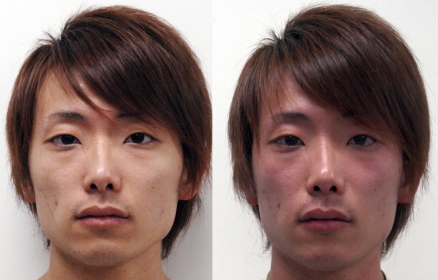
The Alcohol Flushing Response Facial flushing in a 22-year-old ALDH2 heterozygote before (left) and after (right) drinking alcohol. The individual pictured in this figure has given written consent for publication of his picture using the PLoS consent form.

Our goal in writing this article is to inform doctors firstly that their ALDH2-deficient patients have an increased risk for esophageal cancer if they drink moderate amounts of alcohol, and secondly that the alcohol flushing response is a biomarker for ALDH2 deficiency. Because of the intensity of the symptoms, most people who have the alcohol flushing response are aware of it. Therefore clinicians can determine ALDH2 deficiency simply by asking about previous episodes of alcohol-induced flushing. As a result, ALDH2-deficient patients can then be counseled to reduce alcohol consumption, and high-risk patients can be assessed for endoscopic cancer screening. Based on the sizes of the Japanese, Chinese, and Korean populations and the expected frequency of ALDH2-deficient individuals in each [1], we estimate that there are at least 540 million ALDH2-deficient individuals in the world, representing approximately 8% of the population. In a population of this size, even a small reduction in the incidence of esophageal cancer could result in a substantial reduction in esophageal cancer deaths worldwide.

**Summary Points**

- ALDH2 eficiency resulting from the *ALDH2* Lys487 allele contributes to both the alcohol flushing response and an elevated risk of squamous cell esophageal cancer from alcohol consumption.
- Knowledge of the flushing response is useful clinically, as it allows doctors to identify their ALDH2-deficient patients in a simple, cost-effective, and non-invasive manner.
- Doctors should counsel their ALDH2-deficient patients to limit alcohol consumption and thereby reduce the risk of developing esophageal cancer.
- In view of the approximately 540 million ALDH2-deficient individuals in the world, many of whom now live in Western societies, even a small percent reduction in esophageal cancers due to a reduction in alcohol drinking would translate into a substantial number of lives saved.

## A Primer on the Genetics of Alcohol Metabolism

**Figure 2:**
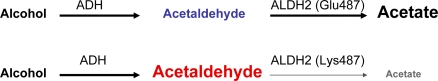
The Ethanol Metabolic Pathway and the Role of the ALDH2 Variants in Acetaldehyde Accumulation It should be noted that ADH is also polymorphic, and genetic variants in ADH1B interact with the ALDH2 variant to modify risk [13].

Ethanol is first metabolized primarily by alcohol dehydrogenase (ADH) into acetaldehyde (Figure 2), a mutagen and animal carcinogen that causes DNA damage and has other cancer-promoting effects [5–7]. Acetaldehyde is subsequently metabolized to acetate, mainly by the enzyme ALDH2 [8]. In East Asian populations there are two main variants of *ALDH2*, resulting from the replacement of glutamate (Glu) at position 487 with lysine (Lys) [9]. The Glu allele (also designated ALDH2*1) encodes a protein with normal catalytic activity, whereas the Lys allele (ALDH2*2) encodes an inactive protein. As a result, Lys/Lys homozygotes have no detectable ALDH2 activity. Because the Lys allele acts in a semi-dominant manner, ALDH2 Lys/Glu heterozygotes have far less than half of the ALDH2 activity of Glu/Glu homozygotes; in fact, the reduction in ALDH2 activity in heterozygotes is more than 100-fold [8].

Alcohol consumed by ALDH2-deficient individuals is metabolized to acetaldehyde, which accumulates in the body due to absent ALDH2 activity and results in facial flushing (Figure 1), nausea, and tachycardia [2]. These unpleasant effects are the result of diverse actions of acetaldehyde in the body, including histamine release [10]. Because of the intensity of this unpleasant response, ALDH2 Lys/Lys homozygotes are unable to consume significant amounts of alcohol. As a result, they are *protected* against the increased risk of esophageal cancer from alcohol consumption [11]. This observation also provided evidence for a causative role for ethanol in esophageal cancer, and a key role for acetaldehyde in mediating this effect [11].

ALDH2 Lys/Glu heterozygotes experience a less severe manifestation of the flushing response due to residual but low ALDH2 enzyme activity in their cells. As a result, some are able to develop tolerance to acetaldehyde and the flushing response and become habitual heavy drinkers, due in part to the influence of societal and cultural factors (see below). Therefore, paradoxically, it is the more common low-activity ALDH2 heterozygous genotype that is associated with greatest risk of esophageal cancer from drinking alcohol.

## Evidence That ALDH2 Deficiency Increases the Risk of Alcohol-Related Squamous Cell Esophageal Cancer

**Figure 3:**
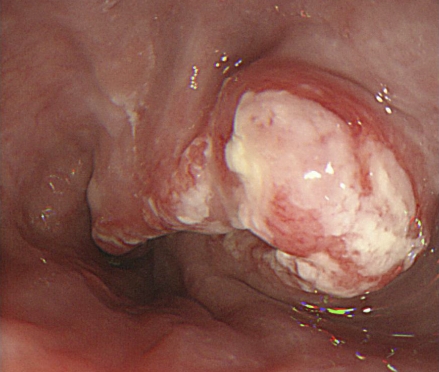
A Late-Stage Squamous Cell Carcinoma of the Esophagus in a 51-Year-Old Male with a Known History of Flushing and Alcohol Drinking

Following the first study [12], which was conducted in the Japanese population, case control studies in Japan and Taiwan have consistently demonstrated a strong link between the risk of esophageal squamous cell carcinoma (Figure 3) and alcohol consumption in low-activity ALDH2 heterozygotes, with odds ratios (ORs) ranging from 3.7 to 18.1 after adjustment for alcohol consumption. Moreover, most studies show ORs of over 10 for increased risk in heterozygotes who are heavy drinkers [13,14]. An independent meta-analysis has also confirmed an increased risk, even among moderate drinking heterozygotes [11]. In the Japanese and Taiwanese studies, a strikingly high proportion (58%–69%) of the excessive risk for esophageal cancer is attributable to drinking by low-activity ALDH2 heterozygous individuals [13,14].

Consistent with the results of case control studies, prospective studies in cancer-free alcoholics have also shown that the relative hazard for future upper aerodigestive tract (UADT) cancers in low-activity ALDH2 heterozygotes is approximately 12 times higher than in individuals with active ALDH2 [15]. (The UADT includes the oral cavity, pharynx, larynx, and esophagus.) In addition, alcohol consumption in low-activity ALDH2 heterozygotes has been associated with other cancer-related outcomes, including the presence of multiple areas of esophageal dysplasia (i.e., premalignant lesions) and multiple independent UADT cancers [13].

It is important to note that ALDH2 deficiency does not influence esophageal cancer risk in non-drinkers [11]. Furthermore, the magnitude of the ALDH2-associated esophageal cancer risk depends on the relative importance of alcohol versus other risk factors in a given population. In rural areas of China, where there is a high rate of esophageal cancer but alcohol drinking plays a less important role than in Japan and Taiwan, there is a more modest positive association (ORs, 1.7 to 3.1) between low-activity ALDH2 heterozygotes and esophageal cancer risk (e.g., [16]).

## Acetaldehyde Is Responsible for Facial Flushing and Esophageal Cancer Risk in ALDH2-Deficient Individuals

**Figure 4:**
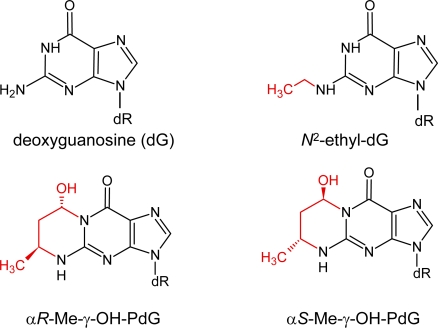
Chemical Structures of Deoxyguanosine, the DNA Base That Is the Target for Acetaldehyde, As Well As the Acetaldehyde-Derived DNA Lesions Referred To in Table 1 The atoms in red represent the acetaldehyde-derived chemical modifications.

Acetaldehyde is responsible for the facial flushing and other unpleasant effects that ALDH2-deficient individuals experience when they drink alcohol [10]. Importantly, there is now direct evidence that ALDH2-deficient individuals experience higher levels of acetaldehyde-related DNA and chromosomal damage than individuals with fully active ALDH2 when they consume equivalent amounts of alcohol, providing a likely mechanism for the increased cancer risk. A study in Japanese alcoholics [17] showed that the amount of mutagenic acetaldehyde-derived DNA adducts (Figure 4) in white blood cells was significantly higher in ALDH2-deficient heterozygotes than in individuals with active ALDH2 (Table 1). In this study, while the two groups were matched for alcohol consumption, the ALDH2-deficient group consumed slightly less alcohol on average than the controls. Also, ALDH2 heterozygotes who drank alcohol had higher levels of white blood cells with chromosomal damage than drinkers with active ALDH2 [18]. Because of these as well as other data, the 2007 International Agency for Research on Cancer Working Group on alcohol and cancer specifically noted the substantial mechanistic evidence supporting a causal role for acetaldehyde in alcohol-related esophageal cancer [19].

**Table 1:**
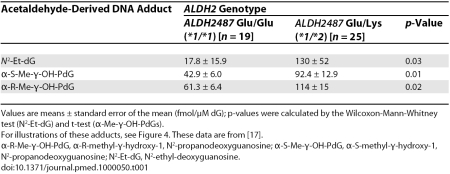
Acetaldehyde-Derived DNA Damage in ALDH2-Deficient Alcoholics

**Five Key Papers in the Field**

**Harada et al., 1981** [2] The first documentation of the relationship between ALDH deficiency and the flushing reaction.

**Yoshida et al., 1984** [9] Identification of the amino acid variant responsible for ALDH deficiency.

**Yokoyama et al., 1996** [12] The first evidence demonstrating that ALDH2-deficient individuals have a dramatically elevated risk of esophageal cancer when they drink alcohol.

**Yokoyama et al., 2003** [25] Demonstrates that an updated flushing questionnaire containing two simple questions is approximately 90% sensitive and specific for identifying ALDH2-deficient individuals.

**Baan et al., 2007** [19] Summary of the conclusions from the 2007 International Agency for Research on Cancer Working Group on the Consumption of Alcoholic Beverages. This is the first report to conclude that ethanol in alcoholic beverages is carcinogenic to humans. The report also adds the female breast and colorectum to the list of sites for alcohol-related carcinogenesis and notes substantial mechanistic evidence linking acetaldehyde to esophageal cancer risk based on studies from ALDH2-deficient individuals.

While the UADT is exposed to acetaldehyde from alcoholic beverages [20] and tobacco smoke, increasing evidence points to the metabolism of ethanol by microorganisms in the oral cavity as an important source of acetaldehyde in saliva and, by extension, in the esophagus. Acetaldehyde levels in saliva are 10–20 times higher than in blood, due to the local formation of acetaldehyde by oral microorganisms [21]. Importantly, ALDH2 heterozygotes had two to three times the acetaldehyde levels in their saliva compared to fully active ALDH2 individuals after a moderate dose of oral ethanol [22].

## Social and Cultural Factors Modulate Alcohol Drinking by ALDH2 Heterozygotes

Alcohol consumption is a social activity, and as such can be strongly influenced by cultural and social forces. In Japan, where the risk of alcohol-related esophageal cancer in ALDH2 heterozygotes has been most well documented, going out drinking after work with colleagues is an essential element of Japanese business society, and the idea of group harmony is particularly powerful. The percentage of heavy drinking men who are low-activity ALDH2 heterozygotes has risen substantially in the last few decades, in parallel with the proliferation of business society in Japan and increases in per capita alcohol consumption. Harada et al. [23] first reported that the frequency of inactive ALDH2 was very low (only 2%) in Japanese alcoholics in 1982. In a later study using archival DNA samples, Higuchi et al. [24] determined that in 1979, 3% of Japanese alcoholics were ALDH2 heterozygotes, compared with 8% in 1986 and 13% in 1992. In a more recent study, approximately 26% of heavy drinking (consuming more than about 400 g of ethanol per week) men in Tokyo were ALDH2 Lys487 heterozygotes [35]. In other East Asian countries, estimates of the percentage of alcoholics who are low-activity ALDH2 heterozygotes range from 17% in Taiwan in 1999 [26] to 4% in Korea in 2007 [27]. Taken together, these observations indicate that the inhibitory effect of heterozygous ALDH2-deficiency on alcohol consumption can be strongly influenced by local social and cultural factors which may change over time.

There are many East Asians now living in Western societies, particularly at universities and in metropolitan areas. A sub-population of special concern is ALDH2-deficient university students who may face peer pressure for heavy drinking and binge drinking. Furthermore, anecdotal evidence indicates that some young people view the facial flushing response as a cosmetic problem and use antihistamines in an effort to blunt the flushing while continuing to drink alcohol [28]. This practice is expected to increase the likelihood of developing esophageal cancer.

## Education and Early Detection Can Reduce the Global Health Burden of Esophageal Cancer

Clinicians who treat patients of East Asian descent need to be aware of the risk of esophageal cancer from alcohol consumption in their ALDH2-deficient patients. Importantly, clinicians can determine whether an individual of East Asian descent is ALDH2 deficient simply by asking whether they have experienced the alcohol flushing response. In the Japanese population, ALDH2 deficiency can be identified accurately based on the answers to a flushing questionnaire consisting of two questions (see Box 1) about previous episodes of facial flushing after drinking alcohol [25]. The two questions can be easily included as part of a standard clinical interview. In a Japanese male population, the flushing questionnaire had a 90% sensitivity and 88% specificity [25] and a positive predictive value of 87% (based on the tabulated data in [25]). The flushing questionnaire gave a similarly high sensitivity (88%) and specificity (92%) when administered to Japanese women [29].

**Box 1. Clinical Tests To Assess ALDH2 Deficiency Due To the *ALDH2* Lys487 Allele**

**1. The Flushing Questionnaire**

The flushing questionnaire consists of two questions: (A) Do you have a tendency to develop facial flushing immediately after drinking a glass (about 180 ml) of beer?; (B) Did you have a tendency to develop facial flushing immediately after drinking a glass of beer in the first one or two years after you started drinking? For both questions, the choice of answers are: yes, no, or unknown.

If an individual answers yes to either question A or B, they are considered to be ALDH2 deficient [25]. The addition of question B is important because some individuals can become tolerant to the facial flushing effect.

The questionnaire that was tested referred to a small (about 180 ml) glass of beer. However, it seems likely that similar results would be obtained if the question were asked about beer or other beverages containing a similar amount of alcohol (about two-thirds of a glass of wine or shot of hard liquor).

**2. The Ethanol Patch Test**

The ethanol patch test is performed as follows: 0.1 ml of 70% ethanol is pipetted onto a 15 × 15 mm lint pad fixed on an adhesive tape. The patch is attached to the inner surface of the upper arm for a 7-minute period and then removed. A patch area that shows erythema 10–15 minutes after removal is judged as positive. The sensitivity, specificity, and positive predictive value for inactive ALDH2 are more than 90% in Japanese youth [34].

**Figure 5:**
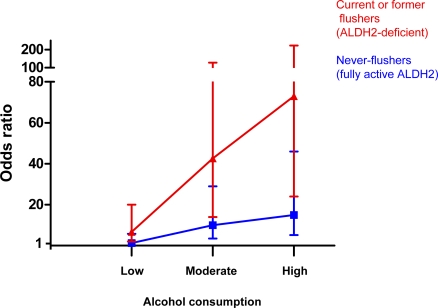
Odds Ratios for Esophageal Cancer at Different Amounts of Alcohol Consumption In Relation To the Flushing Response Alcohol consumption amounts: low, 1–8.9 units/week; moderate, 9–17.9 units/week; high, ≥18 units/week; where 1 unit = 22 g of ethanol. The referent (OR = 1) is never/rare drinkers (<1 unit/week) of either genotype. Odds ratios were adjusted for age, frequency of drinking strong alcohol beverages, pack-years of smoking, and intake of fruit and green-yellow vegetables, based on a multiple logistic regression model. Error bars are 95% confidence intervals. The graph is based on the data in [25].

Once ALDH2-deficient patients have been identified, they should be informed about their elevated risk of developing esophageal cancer risk from drinking alcohol. As can be seen from Figure 5, ALDH2 deficiency increases esophageal cancer risk at all three drinking levels, but the slope of the line relating alcohol consumption to esophageal cancer risk is steeper in ALDH2-deficient individuals. Clinicians might therefore use this graph to explain the increased risk when counseling their ALDH2-deficient patients to reduce alcohol consumption.

The ORs in Figure 5 are adjusted for smoking. However, patients should also be informed that smoking further increases the esophageal cancer risk in a synergistic manner with alcohol [30]. As noted above, cigarette smoking dramatically increases acetaldehyde levels in saliva, and ALDH2-deficient individuals have a reduced capacity to clear salivary acetaldehyde.

For patients at high risk of esophageal cancer, doctors should also consider endoscopy for early cancer detection. A health risk assessment tool to select candidates for endoscopic cancer screening, including data on alcohol flushing as well as alcohol consumption, smoking, and dietary habits, is currently being developed and validated [31]. Using a version of the health risk assessment that includes the flushing questionnaire as a major component, it has been estimated that approximately 58% of esophageal cancers in the Japanese population could be detected by screening only the individuals with the top 10% risk scores [31].

When detected early, esophageal cancer can be treated by endoscopic mucosectomy, a standard and relatively non-invasive procedure. However, once the cancer has grown large enough to penetrate the submucosal layer, the likelihood of lymph node metastasis increases significantly [32]. Only about 20% of esophageal cancer patients survive three years after diagnosis [3], emphasizing the importance of disease prevention.

ALDH2-deficient university students may have their first experiences with heavy drinking while at university. Therefore, it is particularly important for university health professionals to be aware of the relationship between ALDH2 deficiency, facial flushing, and alcohol-related cancer risk. Informing ALDH2-deficient young people of their risk of esophageal cancer from alcohol drinking represents a valuable opportunity for cancer prevention. However, most of the data on the accuracy of the flushing questionnaire have come from individuals over 40 years old. To assess ALDH2 deficiency in young people with little experience of alcohol consumption, an ethanol patch test (see Box 1) can be used [13]. In the patch test, ethanol is applied to the skin, where it is metabolized to acetaldehyde. (Both ADH and ALDH can be detected in skin fibroblasts [33].) If the acetaldehyde is not further metabolized to acetate, it causes vasodilation, which is detected visually as localized erythema. Like the flushing questionnaire, the ethanol patch test is simple and inexpensive to perform, and the sensitivity, specificity, and positive predictive value for inactive ALDH2 have been shown to be more than 90% in Japanese youth [34].

## How Many Cancers Could Be Prevented by Reducing Alcohol Consumption in ALDH2-Deficient Individuals?

Finally, it is important to consider how many esophageal cancer cases might be prevented if ALDH2-deficient individuals reduced alcohol consumption. To address this question, the tabulated data of [35] were used to recalculate the population-attributable risk by Bruzzi's method [36]. The results of this calculation indicate that if moderate or heavy drinking ALDH2 heterozygotes were instead only light drinkers, 53% of esophageal squamous cell carcinomas might be prevented in the Japanese male population.

## Supporting Information

Alternative Language Summary S1 Translation of the article summary into Japanese by AY. (23 KB DOC).

## Abbreviations

ADH: alcohol dehydrogenase
ALDH2: aldehyde dehydrogenase 2
Glu: glutamate
Lys: lysine
OR: odds ratio
UADT: upper aerodigestive tract

## References

[pmed-1000050-b001] Eng MY, Luczak SE, Wall TL (2007). ALDH2, ADH1B, and ADH1C genotypes in Asians: A literature review. Alcohol Res Health.

[pmed-1000050-b002] Harada S, Agarwal DP, Goedde HW (1981). Aldehyde dehydrogenase deficiency as cause of facial flushing reaction to alcohol in Japanese. Lancet.

[pmed-1000050-b003] Umar SB, Fleischer DE (2008). Esophageal cancer: Epidemiology, pathogenesis and prevention. Nat Clin Pract Gastroenterol Hepatol.

[pmed-1000050-b004] Foundation for Promotion of Cancer Research (2008). International comparisons of cancer survival rates. Comparisons of 5-year relative survivals by site—6 Cancer Registries in Japan, the US SEER Program, and Eurocare-4. Cancer Statistics in Japan—2008.

[pmed-1000050-b005] Dellarco VL (1988). A mutagenicity assessment of acetaldehyde. Mutat Res.

[pmed-1000050-b006] Brooks PJ, Theruvathu JA (2005). DNA adducts from acetaldehyde: Implications for alcohol-related carcinogenesis. Alcohol.

[pmed-1000050-b007] Seitz HK, Stickel F (2007). Molecular mechanisms of alcohol-mediated carcinogenesis. Nat Rev Cancer.

[pmed-1000050-b008] Crabb DW, Matsumoto M, Chang D, You M (2004). Overview of the role of alcohol dehydrogenase and aldehyde dehydrogenase and their variants in the genesis of alcohol-related pathology. Proc Nutr Soc.

[pmed-1000050-b009] Yoshida A, Huang IY, Ikawa M (1984). Molecular abnormality of an inactive aldehyde dehydrogenase variant commonly found in Orientals. Proc Natl Acad Sci U S A.

[pmed-1000050-b010] Eriksson CJ (2001). The role of acetaldehyde in the actions of alcohol (update 2000). Alcohol Clin Exp Res.

[pmed-1000050-b011] Lewis SJ, Smith GD (2005). Alcohol, ALDH2, and esophageal cancer: A meta-analysis which illustrates the potentials and limitations of a Mendelian randomization approach. Cancer Epidemiol Biomarkers Prev.

[pmed-1000050-b012] Yokoyama A, Muramatsu T, Ohmori T, Higuchi S, Hayashida M (1996). Esophageal cancer and aldehyde dehydrogenase-2 genotypes in Japanese males. Cancer Epidemiol Biomarkers Prev.

[pmed-1000050-b013] Yokoyama A, Omori T (2003). Genetic polymorphisms of alcohol and aldehyde dehydrogenases and risk for esophageal and head and neck cancers. Jpn J Clin Oncol.

[pmed-1000050-b014] Lee CH, Lee JM, Wu DC, Goan YG, Chou SH (2008). Carcinogenetic impact of ADH1B and ALDH2 genes on squamous cell carcinoma risk of the esophagus with regard to the consumption of alcohol, tobacco and betel quid. Int J Cancer.

[pmed-1000050-b015] Yokoyama A, Omori T, Yokoyama T, Sato Y, Mizukami T (2006). Risk of squamous cell carcinoma of the upper aerodigestive tract in cancer-free alcoholic Japanese men: An endoscopic follow-up study. Cancer Epidemiol Biomarkers Prev.

[pmed-1000050-b016] Yang SJ, Wang HY, Li XQ, Du HZ, Zheng CJ (2007). Genetic polymorphisms of ADH2 and ALDH2 association with esophageal cancer risk in southwest China. World J Gastroenterol.

[pmed-1000050-b017] Matsuda T, Yabushita H, Kanaly RA, Shibutani S, Yokoyama A (2006). Increased DNA damage in ALDH2-deficient alcoholics. Chem Res Toxicol.

[pmed-1000050-b018] Ishikawa H, Ishikawa T, Yamamoto H, Fukao A, Yokoyama K (2007). Genotoxic effects of alcohol in human peripheral lymphocytes modulated by ADH1B and ALDH2 gene polymorphisms. Mutat Res.

[pmed-1000050-b019] Baan R, Straif K, Grosse Y, Secretan B, El Ghissassi F (2007). Carcinogenicity of alcoholic beverages. Lancet Oncol.

[pmed-1000050-b020] Lachenmeier DW, Sohnius EM (2008). The role of acetaldehyde outside ethanol metabolism in the carcinogenicity of alcoholic beverages: Evidence from a large chemical survey. Food Chem Toxicol.

[pmed-1000050-b021] Homann N, Tillonen J, Meurman JH, Rintamaki H, Lindqvist C (2000). Increased salivary acetaldehyde levels in heavy drinkers and smokers: A microbiological approach to oral cavity cancer. Carcinogenesis.

[pmed-1000050-b022] Vakevainen S, Tillonen J, Agarwal DP, Srivastava N, Salaspuro M (2000). High salivary acetaldehyde after a moderate dose of alcohol in ALDH2-deficient subjects: Strong evidence for the local carcinogenic action of acetaldehyde. Alcohol Clin Exp Res.

[pmed-1000050-b023] Harada S, Agarwal DP, Goedde HW, Tagaki S, Ishikawa B (1982). Possible protective role against alcoholism for aldehyde dehydrogenase isozyme deficiency in Japan. Lancet.

[pmed-1000050-b024] Higuchi S, Matsushita S, Imazeki H, Kinoshita T, Takagi S (1994). Aldehyde dehydrogenase genotypes in Japanese alcoholics. Lancet.

[pmed-1000050-b025] Yokoyama T, Yokoyama A, Kato H, Tsujinaka T, Muto M (2003). Alcohol flushing, alcohol and aldehyde dehydrogenase genotypes, and risk for esophageal squamous cell carcinoma in Japanese men. Cancer Epidemiol Biomarkers Prev.

[pmed-1000050-b026] Chen CC, Lu RB, Chen YC, Wang MF, Chang YC (1999). Interaction between the functional polymorphisms of the alcohol-metabolism genes in protection against alcoholism. Am J Hum Genet.

[pmed-1000050-b027] Kim DJ, Choi IG, Park BL, Lee BC, Ham BJ (2008). Major genetic components underlying alcoholism in Korean population. Hum Mol Genet.

[pmed-1000050-b028] Haynie D (2007 April 25). Asian party-goers strive to get rid of that ‘glow’. http://www.azcentral.com/ent/pop/articles/0425asianglow-CR.html.

[pmed-1000050-b029] Yokoyama A, Kato H, Yokoyama T, Igaki H, Tsujinaka T (2006). Esophageal squamous cell carcinoma and aldehyde dehydrogenase-2 genotypes in Japanese females. Alcohol Clin Exp Res.

[pmed-1000050-b030] Lee CH, Wu DC, Lee JM, Wu IC, Goan YG (2007). Carcinogenetic impact of alcohol intake on squamous cell carcinoma risk of the oesophagus in relation to tobacco smoking. Eur J Cancer.

[pmed-1000050-b031] Yokoyama T, Yokoyama A, Kumagai Y, Omori T, Kato H (2008). Health risk appraisal models for mass screening of esophageal cancer in Japanese men. Cancer Epidemiol Biomarkers Prev.

[pmed-1000050-b032] Bollschweiler E, Baldus SE, Schroder W, Prenzel K, Gutschow C (2006). High rate of lymph-node metastasis in submucosal esophageal squamous-cell carcinomas and adenocarcinomas. Endoscopy.

[pmed-1000050-b033] Goedde HW, Agarwal DP, Harada S (1979). Alcohol metabolizing enzymes: Studies of isozymes in human biopsies and cultured fibroblasts. Clin Genet.

[pmed-1000050-b034] Muramatsu T, Higuchi S, Shigemori K, Saito M, Sasao M (1989). Ethanol patch test—A simple and sensitive method for identifying ALDH phenotype. Alcohol Clin Exp Res.

[pmed-1000050-b035] Yokoyama A, Kato H, Yokoyama T, Tsujinaka T, Muto M (2002). Genetic polymorphisms of alcohol and aldehyde dehydrogenases and glutathione S-transferase M1 and drinking, smoking, and diet in Japanese men with esophageal squamous cell carcinoma. Carcinogenesis.

[pmed-1000050-b036] Bruzzi P, Green SB, Byar DP, Brinton LA, Schairer C (1985). Estimating the population attributable risk for multiple risk factors using case-control data. Am J Epidemiol.

