# Supplementary material for: The Alcohol Flushing Response: An Unrecognized Risk Factor for Esophageal Cancer from Alcohol Consumption
Source: PLoS Med. 2009 Mar 24;6(3):e1000050. doi: 10.1371/journal.pmed.1000050 (PMC2659709; doi:10.1371/journal.pmed.1000050)
Supplement: Supplementary file 1 [file pmed.1000050.sd001.doc]

ALDH2 Lys487allele によるALDH2欠損は、飲酒による顔が赤くなるフラッシング反応と食道扁平上皮癌のリスク上昇の原因となる。フラッシング反応についての知識は臨床上有用で、医師がコストをかけずに簡単にALDH2欠損者を特定するのに役立つ。その結果、医師はALDH2欠損者に飲酒量を制限することを助言でき、食道癌発生のリスクを減らすことができる。世界には約5億4千万人のALDH2欠損者がおり、現在、欧米社会で暮らしているひとも多い。したがって飲酒を減らすことによる食道癌の減少率が少ないとしても全体としてかなり多くの生命を救うことになるだろう。
